# KRIPO – a structure-based pharmacophores approach explains polypharmacological effects

**DOI:** 10.1186/1758-2946-6-S1-O26

**Published:** 2014-03-11

**Authors:** Tina Ritschel, Tom JJ Schirris, Frans GM Russel

**Affiliations:** 1 Computational Discovery and Design (CDD) Group at the Centre for Molecular and Biomolecular Informatics (CMBI),UMC Nijmegen, The Netherlands; 2 Dept. of Pharmacology and Toxicology, UMC, Nijmegen, the Netherlands; 3 Centre for Systems Biology and Bioenergetics, UMC, Nijmegen, the Netherlands

## 

“The most fruitful basis for the discovery of a new drug is to start with an old one” is a citation from Sir James Black’s Nobel laureate (1988).

The background of this statement lies in the fact that most drugs are able to bind to multiple protein targets in the human body, this is known as polypharmacology. This behaviour can lead to unwanted side effects, and innovative research to avoid such adverse properties is of great importance. Paradoxically, polypharmacology can also be used to create new therapeutic approaches, as the protein to which a drug binds causing a side effect in one case, can be the main target for another treatment. Many cases report about the problems and opportunities of polypharmacology.

## Aims

In order for a drug to bind to multiple targets, the interaction sites of these targets must be similar on a molecular level. Using KRIPO (Key Representation of Interaction in POckets) [1] with specially developed pharmacophore fingerprints, we provide an objective method to accurately describe protein interactions.

## Results

KRIPO was used to explain the molecular mechanism of adverse drug effects of HMG-CoA reductase inhibitors, better known as statins. A previously unknown binding site for statins in cytochrome b, the major subunit of mitochondrial complex III of the oxidative phosphorylation system, was predicted by KRIPO.

## Conclusion

Combining docking studies with KRIPO and experimental data on complex III inhibition enabled us to explain the molecular details of statin binding to the predicted binding site.
